# How Health Care Providers Can Use Digital Health Technologies to Inform Human Papillomavirus (HPV) Decision Making and Promote the HPV Vaccine Uptake Among Adolescents and Young Adults

**DOI:** 10.1089/biores.2018.0051

**Published:** 2019-06-10

**Authors:** Versie Johnson-Mallard, Gabrielle Darville, Rebeccah Mercado, Charkarra Anderson-Lewis, Jann MacInnes

**Affiliations:** ^1^ Department of Family, Community and Health System Science, University of Florida, Gainesville, Florida.; ^2^ Department of Academic Affairs, College of Public Health, University of Georgia, Athens, Georgia.; ^3^ Department of Pediatrics in the College of Medicine, University of Florida, Gainesville, Florida.; ^4^ Department of Public Health, University of Southern Mississippi, Hattiesburg, Mississippi.; ^5^ Department of Human Development and Organization Studies in Education, College of Education, University of Florida, Gainesville, Florida.

**Keywords:** HPV, preventive health, vaccine

## Abstract

High-risk stains of human papillomavirus (HPV) is linked to causing cancer, is highly prevalent, and has increased incidence among adolescents and young adults. However, vaccination rates are low. Health care provider recommendation is the biggest influencer toward vaccine uptake. Since more health care providers are using digital health technologies in their medical practices, this study investigated the feasibility of technology to increase informed decision making. A convenience sample of 210 students completed an online survey. Participants were 18–25 years of age (88%), female (85%), Caucasian (60%), and never been diagnosed with HPV (92.9%). Overwhelmingly, participants owned a smartphone (98.9%) and used mobile apps for health/health tracking (65.5%). However, only 29.3% indicated that they received text messages from their health care provider. Digital health technology can be a cost-effective way for increasing HPV knowledge, removing barriers, and increasing vaccine uptake. Health care providers should explore using various platforms to empower their health care decision making.

## Introduction

Human papillomavirus (HPV) infection is the most common sexually transmitted infection (STI) in the United States and is responsible for 99% of all cervical cancer cases worldwide.^1,2^ HPV is also associated with other forms of cancer such as anal, oropharyngeal, vaginal, vulvar, and penile.^3,4^ Although vaccines are available to reduce the risk of infection by several of the most prevalent strains of this virus, immunization rates continue to remain low. The HPV vaccine is a two-dose regimen, a shift from a three-dose regimen in 2016, offering protection to those not completing the HPV vaccine series. Specifically, a two-dose regimen is recommended for girls and boys starting the HPV series at ages 9 through 14 years, three doses remain for boys and girls starting the HPV series at ages 15 through 26 years. In October 2018, the Food and Drug Administration (FDA) expanded the approved use of the HPV vaccine (Gardasil-9) to include women and men ages 27 through 45 years.^5^ In data from the 2016 National Immunization Survey, only 65% of adolescent females and 56% of males initiated the two- or three-dose vaccine series with 50% of adolescent females and 38% of males completing the series.^6^ Not only are these rates lower than the Healthy People 2020 goal of 80% vaccination, but there is also a distinct difference in the number of adolescent males being vaccinated compared with females.^7,8^ This disparity may not only be due, in part, to the 5-year lag in the Centers for Disease Control and Prevention (CDC)'s recommendation for the HPV vaccine in adolescent males (2011) compared with adolescent females (2006), but it may also be attributed to the “feminization of HPV” as a women's problem emphasizing the need for frequent and early recommendations by health care providers to parents of adolescent males in particular.^9^

Current literature highlights the importance of health care providers in increasing vaccine uptake with the most common correlate of HPV vaccination acceptance being a provider's recommendation and the most commonly cited reason for nonvaccination being a lack of provider recommendation.^10^ In a nationally representative survey of parents, those parents with adolescent males more frequently expressed being unsure or not likely to have their child vaccinated against HPV, compared with parents of adolescent females, primarily due to a lack of provider recommendation or knowledge. If providers regularly inform patients and parents about the benefits of the HPV vaccine as a cancer prevention, particularly those who are unsure, as well as distribute reminders to patients who are due for vaccination based on recommended immunization schedules, there is an opportunity to improve HPV vaccine uptake among adolescents.^11,12^

One way providers can better inform patients and parents as well as provide reminders of upcoming immunizations is through the use of technology. Text messaging and smartphone applications (apps) present two areas in new media with potential for use as vaccine uptake interventions. Both technologies are highly accessible and popular worldwide, and their usage is more evenly distributed across divisions of race, class, and international borders compared with other devices and services such as desktop and laptop computers and home broadband.^13–15^

Current literature indicates an increase in the use of text messaging and apps as mobile health interventions, with evidence that these methods have succeeded in reducing missed appointments, educating adolescents about sexual health, and tracking patient information. However, very little existing research has examined the application of these technologies to interventions for HPV vaccine uptake and completion of the two- or three-dose series, demonstrating the need for further inquiry within this area.

## Literature Review

### HPV pathology and epidemiology

HPV refers to a group of over 150 related types of a DNA tumor virus that causes abnormal growth and accumulation of epithelial tissue in skin and mucous membranes,^1,16^ More than 40 types of HPV infect the human genital tract and can be transmitted through direct sexual contact, including oral, vaginal, and anal sex. Each of these sexually transmitted types of HPV falls into one of two categories: nononcogenic or low-risk strains that can cause the formation of skin warts in the anogenital area and the oncogenic or high-risk strains that can cause cervical, anal, oropharyngeal, vaginal, vulvar, and penile cancers.^17^

HPV is responsible for ∼90% of all cases of genital warts, 99% of all cervical cancers, 95% of all anal cancers, and 70% of all oropharyngeal cancers worldwide.^1,2,17^ HPV is the most common STI in the United States, with ∼79 million Americans currently infected and 14 million becoming newly infected every year. The global prevalence of HPV infection in women without cervical abnormalities is ∼11% to 12%, with higher regional rates in sub-Saharan Africa, Eastern Europe, and Latin America.^18^ The worldwide prevalence of HPV infection among males receives little to no coverage in the current literature.

### HPV vaccination

To reduce the risk of HPV infection and its potential health effects, the U.S. (CDC) recommends that individuals use latex condoms during sexual activity, attend routine screenings for cervical cancer if applicable, and receive HPV vaccinations.^1^ The CDC currently recommends that all children ages 11 to 12 receive two doses of an HPV vaccine 6 to 12 months apart; “catch-up” vaccination is also recommended on a three-dose schedule for females up to age 26 years, males up to age 21 years, transgender adults up to age 26 years, and young adults with immunocompromising conditions such as HIV/AIDS up to age 26 years. Three noninfectious HPV vaccines are currently approved for use by the U.S. FDA.^5^

The bivalent vaccine Cervarix (2vHPV, manufactured by GlaxoSmithKline in Rixensart, Belgium) targets HPV types 16 and 18, the oncogenic strains responsible for the majority of all HPV-related cancers. The quadrivalent vaccine Gardasil (4vHPV, manufactured by Merck and Co, Inc., in Whitehouse Station, NJ) adds protection from strains 6 and 11, which are responsible for genital warts; its nonavalent counterpart, Gardasil-9 (9vHPV), includes additional protection from types 31, 33, 45, 52, and 58.^19^ In 2015, ∼62.8% of females and 49.8% of males in the United States had received at least one dose of an HPV vaccine; only 41.9% of females and 28.1% of males had attained complete vaccine adherence with three or more doses.^20^ An estimated 59 million women worldwide have received at least one dose, making up 1.7% of the global female population, while 47 million—1.4%—have received the complete course of the vaccine.^21^ Data on international male HPV vaccine coverage are absent from currently available literature.

### Theoretical application

**FIG. 1.**
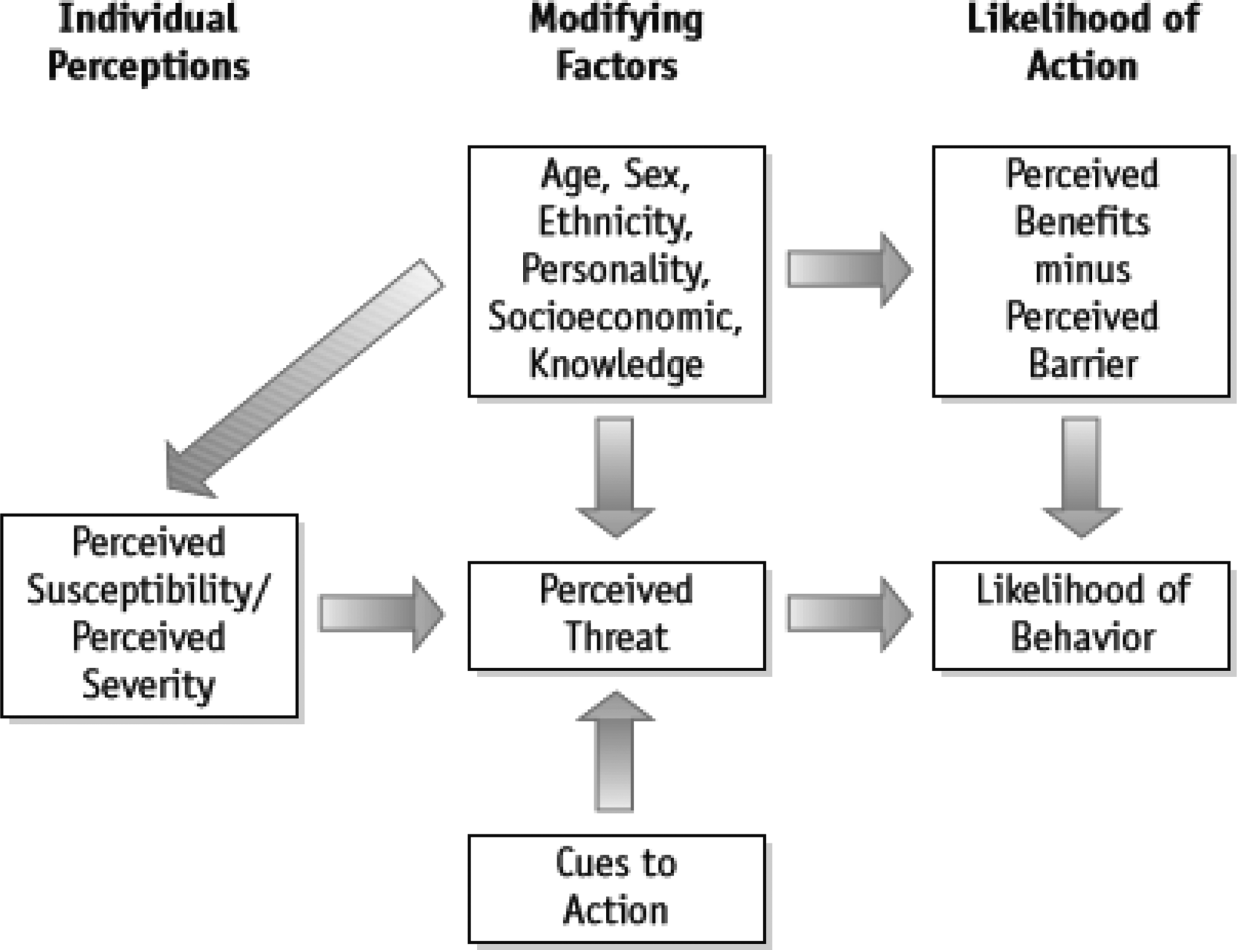
Health belief model.

Digital health technologies, as a cue to action within the health belief model (HBM) as seen in Figure 1, can impact the health decision making among adolescent and newly transitioned college students.^22^ This is especially important when communicating messages about HPV and the HPV vaccine because research has shown that adolescents and college students alike are unaware of their risk of contracting the disease and how the HPV virus can be contracted. Because of this, the purpose of this research study is to determine how health care providers can utilize digital health technologies (such as the internet, mobile phones, short message service [SMS] text messages, and mobile apps) to increase individual risk perception of the HPV virus, increase awareness about the HPV vaccine, overcome barriers to the HPV vaccine, and help adolescents and young adults make informed decisions to get the HPV vaccine. The research questions that guided the research agenda are as follows:

- R01: How knowledgeable are college students of the HPV virus?
- R02: What are the beliefs and attitudes of college students toward the HPV vaccine?
- R03: What barriers exist for college students preventing uptake of the HPV vaccine?
- R04: What digital health platforms (channels) would college students prefer to receive information about the risk of the HPV virus?

## Methodology

### Sample

The study was conducted among both male and female undergraduate and graduate students at a large public university in North Florida. Similar to other interventions focused on college students, they were selected as the study participants because of their constant exposure to the internet and technology. As Patel et al.^23^ explains, research should find ways “to implement HPV interventions via technologies acceptable to college students (e.g., text messaging, e-mail, social networking media) etc.” as it could improve the efficacy of interventions aimed at this population.^23^ Quantitative data on the survey included questions that asked about demographic characteristics, sexual health behaviors, knowledge of HPV, barriers to HPV uptake, and digital health technology practices.

### Procedure

After Institutional Review Board approval was obtained, faculty of undergraduate and graduate courses in the Department of Health Education and Behavior were contacted and data collection was conducted with this convenience sample. Because of the descriptive nature of the survey, no inclusion and exclusion criteria were established. The survey was implemented using online software. Before data collection, the survey was pilot tested with a sample of undergraduate and graduate students (*n* = 81). Feedback provided from this sample was used to revise the survey for data collection, which was conducted over a period of 6 months.

Study participants were provided with an electronic link of the survey from their faculty. This link also included an inform consent page, which provided a disclaimer informing them of the sexual nature of the questions and listed on campus resources should they need to discuss any concerns they may have. Once consent was electronically obtained, participants were promoted to begin the survey. No incentives were offered by researchers for participating in the survey, however, faculty of the courses could offer extra credit if they chose to do so.

### Survey instrument

Using validated and reliable scales found in HPV risk perception research^11,24–26^ and questions about technology use and digital health practices from Pew Research Center's Mobile Health, Health Tracking and Social Media Update Surveys,^27–29^ researchers developed a 54-item questionnaire, the Digital Health Solutions for Informed Decision Survey. Participants could differ in their number of responses per question. Multiple responses were possible. Participants could also decline to answer any question to which they did not want to answer.

Apart from demographical information, students provided answers to items that asked about their sexual activity behavior, sexual intercourse engagement, current sexual activity, number of sexual partners in the past 30 days, total number of lifetime partners, type of sexual activity ever engaged in, contraception use, condom use, gender of all sexual partners, history of sexually transmitted disease (STD) or HPV diagnosis, and HPV vaccination practices. Students answered questions concerning HPV knowledge, sources used to obtain HPV information, and beliefs regarding HPV. Their beliefs as it relates to cancer and HPV vaccination were measured, for this population used the Health Belief Scale, which included dimensions on four constructs of the HBM (perceived severity, perceived susceptibility, perceived benefits, and perceived barriers).

Students were also provided a list of frequently cited barriers throughout HPV literature and asked to select all that apply to them. Lastly, items included questions that also asked about smartphone ownership, smart phone usage, tracking devices/application use, types of health apps, digital health information practices, social media channel frequency use and access routes, SMS health messages, preference of channel to receive information, comfortableness using these digital technologies for HPV and HPV vaccine information, and interest in receiving information through SMS text message or mobile apps. Within this questionnaire, there were several questions included, an “other” response item, to allow participants the opportunity to provide information if the best response was not listed.

### Data analysis

Descriptive statistics (means and frequencies) were calculated for the measures. Because researchers used previously validated and reliable scale items identified in literature, Cronbach' alpha was not calculated for the purpose of this research study.

## Results

Although 210 participants contributed to this research study, survey responses varied for each section of questions as some questions included missing data. All results collected are reported in Tables 1–8. Male and female study participants completed the self-administered survey (Table 1). Demographic characteristics identified that the majority of the population was 18–25 years old (*n* = 147; 88%), female (*n* = 143; 85.6%), not of Hispanic origin (*n* = 136; 64.8%), White/Caucasian (*n* = 126; 60%), upperclassman and seniors (*n* = 103; 61.6%), went to school fulltime (*n* = 154; 92.2%), were Florida residents (*n* = 161; 97.6%), and had private health insurance through their parents (*n* = 122; 73.1%). The majority of the survey respondents were citizens of the United States (Table 1).

**Table 1.** Demographic Variables, Frequency, and Percent

Descriptive variables	Frequency (*n*)	Valid percent (%)	Descriptive variables	Frequency (*n*)	Valid percent (%)
Age (years)			Classification		
18–25	147	88	Freshman (1st year)	33	19.8
26–34	14	8.4	Sophomore (2nd year)	21	12.6
35–54	4	2.0	Upperclassman (3rd year)	61	36.5
45–54	1	0.6	Senior (4th + year)	42	25.1
55–64	1	0.6	Graduate student	10	6.0
Total	167	100	Total	167	100
Gender			Student status		
Male	24	14.4	Part-time	13	7.8
Female	143	85.6	Full-time	154	92.2
Total	167	100	Total	167	100
^*^Ethnicity (check all that apply)			Health insurance		
Not of Hispanic, Latino (a) or Spanish Origin	136	81	Private through parents	122	73.1
Puerto Rican	7	4.0	Work or University Insurance	21	12.6
Cuban	4	2.0	Public or other insurance	16	9.6
Another Hispanic, Latino, or Spanish Origin	15	8.9	None or not sure	8	4.8
Do not wish to respond	7	4.0	Total	167	100
Total	169	100	Nationality/citizenship		
^*^Race (check all that apply)					
Black or African American	14	8			
White or Caucasian	126	75	Canada	1	0.6
Asian Indian	5	3	China	2	1.2
Chinese	4	2	Honduras	1	0.6
Filipino	9	5	Malaysia	1	0.6
Korean	4	2	Pakistan	1	0.6
Vietnamese	3	2	Peru	1	0.6
Do not wish to respond	6	4	Romania	1	0.6
Total	171	100	United Kingdom	1	0.6
Residency (where do you currently live?)			United States	157	94
California	1	0.6	Vietnam	1	0.6
Florida	161	97.6	Total	167	100
Georgia	1	0.6			
New York	1	0.6			
Texas	1	0.6			
Total	165	100			

^*^ *Note:* One or more categories could be selected.

**Table 2.** Sexually Transmitted Diseases and Human Papillomavirus Diagnoses/Vaccine: Frequency and Percent

	**Frequency (*n*)**	**Valid percent (%)**
STD diagnoses		
Yes	19	9.7
No	177	90.3
Total	196	100
HPV diagnoses		
Yes	14	7.1
No	182	92.9
Total	196	100
HPV vaccine received		
No	33	17.3
No, I am outside of the recommended age range	11	5.8
Yes, I have already completed the vaccine series	119	62.3
Yes, I have started the vaccine series (three shots) and intend to complete it	14	7.3
Yes I have started the vaccine and DO NOT intend to complete it	6	3.1
Do not know my HPV vaccination status	8	4.2
Total	191	100
Received doses of the HPV vaccine		
1 dose	7	3.7
2 doses	16	8.4
All 3 doses	117	61.6
None	40	21.1
Do not know my HPV vaccination status	10	5.3
Total	190	100

HPV, human papillomavirus; STD, sexually transmitted disease.

**Table 3.** Human Papillomavirus Knowledge Among College Students

HPV knowledge	Total	Correct answer (*n*)	Valid percentage (%)
1. People with certain HPV types always develop health problems. (False)	185	136	73.5
2. Women can get HPV. (True)	185	184	99.5
3. Condoms effectively protect against HPV infection. (False)	185	82	44.3
4. HPV may spread from person to person by sexual intercourse. (True)	185	185	100
5. Males may be infected with HPV and not know it. (True)	185	180	97.3
6. HPV infection can be cured with antibiotics. (False)	185	155	83.8
7. Men can get HPV. (True)	185	181	97.8
8. If you get HPV, you will have HPV for life. (False)	185	49	26.5
9. Females may be infected with HPV and not know it. (True)	185	183	98.9
10. A person can get HPV by having sex. (True)	185	185	100
11. HPV infection among women is rare. (False)	185	177	95.7
12. Condoms always protect you from HPV. (False)	185	177	95.7
13. HPV may be spread from person to person through oral sex. (True)	184	169	91.8
14. HPV infection among men is rare. (False)	184	145	78.8

**Table 4.** Beliefs Statements: Human Papillomavirus Vaccine by Level of Agreement

Belief statement	Total	Strongly disagree	Disagree	Neither agree or disagree	Agree	Strongly agree
		*n* (%)	*n* (%)	*n* (%)	*n* (%)	*n* (%)
I do not believe in vaccinations generally.	176	122 (69.3)	39 (22.2)	11 (6.3)	3 (1.7)	1 (0.6)
Vaccinations are not effective and do not prevent diseases.	176	132 (75)	34 (19.3)	10 (5.7)	0 (0)	0 (0)
It is not important to receive all vaccinations.	176	101 (57.4)	42 (23.9)	17 (9.7)	8 (4.5)	8 (4.5)
It is preferable to get the diseases and be protected naturally than to vaccinate.	176	118 (67.0)	40 (22.7)	16 (9.1)	1 (0.6)	1 (0.6)
I do not have confidence that the HPV vaccine is safe.	176	90 (51.1)	43 (24.4)	28 (15.9)	12 (6.8)	3 (1.7)
I believe if I receive the HPV vaccine, I will not be protected from cervical cancer (anal/penile cancer if male).	175	71 (40.6)	56 (32.0)	35 (20)	11 (6.3)	2 (1.1)
I believe that if I receive the HPV vaccine, I will not be protected against HPV.	176	80 (45.5)	63 (35.8)	23 (13.1)	6 (3.4)	4 (2.3)
I do not have enough information about HPV.	176	38 (21.6)	37 (21.0)	36 (20.5)	57 (32.4)	8 (4.5)
I do not have enough information about the HPV vaccine.	176	43 (24.4)	37 (21.0)	36 (20.5)	51 (29.0)	9 (5.1)
Cost would influence my uptake of the HPV vaccine.	176	54 (30.7)	40 (22.7)	28 (15.9)	39 (22.2)	15 (8.5)
My insurance/health care provider did not allow me to receive the vaccine.	176	87 (49.4)	39 (22.2)	48 (27.3)	1 (0.6)	1 (0.6)
I did/do not have money for vaccination.	176	78 (44.3)	52 (29.5)	34 (19.3)	9 (5.1)	3 (1.7)
My sexual behavior is safe.	176	6 (3.4)	11 (6.3)	28 (15.9)	63 (35.8)	68 (38.6)
I do not believe that HPV is exceptionally harmful.	176	94 (53.4)	51 (29.0)	14 (8.0)	12 (6.8)	5 (2.8)

**Table 5.** Barriers to Human Papillomavirus Vaccine Uptake

Barrier (s)	Frequency (*n*)	Percent (%)
1. Safety/long-term side effects of the vaccine	22	10.5
2. Cost/vaccine not covered by health insurance	32	15.2
3. Vaccine is painful/discomfort with injection	13	6.2
4. Vaccine coverage (not preventing all HPV strains or all STIs)	10	4.8
5. Not at risk or low risk for HPV	24	11.4
6. Not a priority	25	11.9
7. Access to health care provider	8	3.8
8. Embarrassed, self-conscious, or uncomfortable talking to health care provider	7	3.3
9. Lack of information and knowledge about HPV or HPV vaccine	34	16.2
10. Parental influence in decision-making process (i.e., mother/parents advised not to obtain the vaccine)	23	11.0
11. Have already been diagnosed with HPV	7	3.3
12. Never had sexual intercourse	13	6.2
13. Current contraception practices are sufficient for protection against HPV	3	1.4
14. Outside the recommended age group	14	6.7
15. Other	8	3.8
16. Not applicable, I already got the vaccine	104	49.5

*Note: N* = 210.

STI, sexually transmitted infection

**Table 6.** Current Social Media Usage Trends Among College Students (*N* = 210)

Social media use	Smartphone, *n* (%)	Tablet, *n* (%)	Computer, *n* (%)	Do not use, *n* (%)
Facebook	159 (75.7)	42 (20%)	144 (68.6)	7 (3.3)
Instagram	142 (67.6)	15 (7.1)	24 (11.4)	26 (12.4)
LinkedIn	14 (6.7)	5 (2.4)	29 (13.8)	121 (57.6)
Pinterest	78 (37.1)	20 (9.5)	80 (38.1)	56 (26.7)
Twitter	70 (33.3)	11 (5.2)	31 (14.8)	86 (41.0)
Other	16 (7.6)	1 (0.5)	7 (3.3)	70 (33.3)

Note response range: several times a day, once a day, three times a week, 1–2 days a week, every few weeks, less often, I do not use.

**Table 7.** Current Social Media Visit Frequencies Among College Students

Frequency of visiting social media site	Facebook, *n* (%)	Instagram, *n* (%)	LinkedIn, *n* (%)	Pinterest, *n* (%)	Twitter, *n* (%)	Other, *n* (%)
Several times a day	124 (59)	113 (53.8)	1 (0.5)	17 (8.1)	34 (16.2)	12 (5.7)
About once per day	27 (12.9)	17 (8.1)	5 (2.4)	13 (6.2)	10 (4.8)	2 (1.0)
3–5 times a week	6 (2.9)	8 (3.8)	2 (1.0)	16 (7.6)	8 (3.8)	0 (0)
1–2 days a week	3 (1.4)	1 (0.5)	5 (2.4)	19 (9.0)	4 (1.9)	2 (1.0)
Every few weeks	2 (1.0)	3 (1.4)	12 (5.7)	25 (11.9)	8 (3.8	1 (0.5)
Less often	1 (0.5)	13 (6.2)	19 (9.0)	19 (9.0)	13 (6.2)	0 (0)
I do not use	7 (3.3)	30 (14.3)	123 (58.6)	58 (27.6)	88 (41.9)	107 (51)

**Table 8.** Preferred Digital Health Technologies by College Students to Communicate About Human Papillomavirus (HPV) Risk and the HPV Vaccine

Characteristics	Frequency (*n*)	Valid percent (%)
Channel/platform preferred to receive information about HPV risk		
Internet or online website	127	73.8
Social media	11	6.4
Mobile app	18	10.5
SMS text messages	13	7.6
Digital games	3	1.7
Total	172	100
Do you receive text message updates from your health care provider		
Yes	51	29.3
No	123	70.7
Total	174	100
Interest in receiving health-related text messages on the HPV virus		
Yes	51	29.7
No	121	70.3
Total	172	100
Interest in receiving health-related text messages on the HPV vaccine		
Yes	41	23.8
No	131	76.2
Total	172	100
Frequency of SMS text messages on HPV (risk factors, vaccine information, local resources, and health care provider info)		
Once per week	42	25
Every other day	1	0.6
Set my own schedule/frequency	36	21.4
None, I do not want to receive text messages	89	53
Total	168	100
Information to be included in SMS text messaging on the HPV vaccine		
The safety of the vaccine	67	31.9
Insurance plans that cover the vaccine	48	22.9
Realistic expectations of discomfort of the vaccine	51	24.3
The effectiveness of the vaccine	78	37.1
College student's risk of acquiring HPV	69	32.9
How to talk to a health care provider about acquiring the HPV vaccine	30	14.3
How to access information and knowledge about HPV and the HPV vaccine	51	24.3
How to talk to your parents about sex and the importance of receiving the HPV vaccine	19	9.0
Measures that college students already diagnosed with HPV can take to manage their condition	44	21.0
How college students can negotiate using contraception practices that provide protection against HPV with your partner	33	15.7
Other	1	0.5
None, I do not want to receive text messages	91	43.3
Interest in using an “app” on smartphone on HPV or the HPV vaccine		
Yes	59	34.9
No	110	65.1
Total	169	100
Likeliness to use a health-related app for HPV and the HPV vaccine		
Very unlikely	57	33.9
Unlikely	28	16.7
Somewhat unlikely	18	10.7
Undecided	16	9.5
Somewhat likely	28	16.7
Likely	17	10.1
Very likely	4	2.4
Total	168	100

*Note:* Preference ranged from 1 = internet/online website; 2 = social media; 3 = mobile apps; 4 = SMS text messages; 5 = digital games.

SMS, short message service.

Before answering specific questions related to sexual practices and behavior, HPV risk perception, HPV vaccine beliefs and knowledge, and technology behavior, study participants were asked whether the HPV vaccine should be mandatory for children ages 9–12 years. Overwhelmingly, 74% of students (*n* = 144) out of 195 total responses indicated that the vaccine should be mandatory for adolescent-aged children. Reasons for making HPV vaccinations mandatory at that age are highlighted in the direct quotes noted below:

Participant 1: “The age range was chosen because it is a time before a child's first encounter of sexual intercourse and exposure to HPV. With that being the case, it serves as a great preventative measure for children and I think that it would only benefit the population if it is mandatory. However, I do feel that there should be exceptions to the requirement for those who would not benefit from the vaccine.”
Participant 2: “Because the age kids are still in the routine of getting vaccinated.”
Participant 3: “To children it is just another shot, however it can save their lives in the future. Even if the children do not grow up to be sexually active, it is at least a precaution.”
Participant 4: “I think it should be mandatory because taking the vaccine during the best immune response time would likely have a significant impact on public health outcomes, and although one concern parents may have is that it might encourage an earlier age of initiation for sexual activity, I don't think those two factors are necessarily related since young children don't need to know that the vaccine is for an STI. Being protected from one STI should not be an enabling factor for unsafe sex if there is proper sex education involved (which there always should be).”

### Variables of interest

Responses to STD/HPV vaccine and sexual history behaviors can be found in Tables 1 and 2. Majority of the sample was reproductive age non-Hispanic women (Table 1). Greater than 90% of the study sample self-reported no history of STDs and no history of HPV (Table 2). Greater than 62% received three doses of HPV vaccine. Table 3 highlights the level of HPV awareness and knowledge among our study population. Areas of concern, and questions answered incorrectly included: you get HPV for life, and condoms protect against HPV infection. Safety appears to be a concern for about half of the study population, 51% do not have confidence that the HPV vaccine is safe, and 47% believe the HPV vaccine is harmful (Table 4). Study participants in our sample were queried in greater detail about their beliefs and were asked to indicate their level of agreement concerning 14 statements related to key barriers to HPV vaccine uptake. Lack of information and knowledge about HPV or HPV vaccine was identified as a barrier to HPV uptake (Table 5).

Information concerning study sample current digital health technology practices were queried (Table 6). Most used smartphones to access social media several times a day (Table 7). Facebook was the highest social media trend (∼76%) followed by Instagram (68%) and LinkedIn not being used often (7%; Table 6). When asked about the preferred digital health technologies to communicate about HPV risk and vaccine, the preferred platform was identified as internet or online website. Receiving text messages about HPV was not popular, only ∼29% were interested in receiving health-related text. Comfort with using various health channels to receive HPV vaccine information was low for social network sites (22%), and digital gaming (13.5%; Table 8).

## Discussion

Evidence was explored to support strategies to facilitate digital health technologies aimed at increasing risk perception of the HPV virus and awareness of the HPV vaccine. The preferred method of communication by college students to communicate HPV risk and vaccine was internet or online websites. Strategy, use of the internet, mobile phones, SMS test messages, and mobile apps to help disseminate evidence-based HPV and HPV vaccination information to adolescents and college students was explored. Currently, most college students reported not receiving text messages from health care providers as updated to health care or messages on the HPV virus. In this study, electronic surveys explored preferred digital health technologies in addition to HPV knowledge, individual risk perception, and awareness of the HPV vaccine. The HPV vaccine was approved for use in boys in 2011 and girls in 2006. Knowledge and awareness of HPV and HPV vaccine were significant and high among our study participants. The majority of the study participants were within the age group of HPV vaccination recommendation (i.e., 18–26 years) and eligible to remain on their parent's insurance plans. Nationally, 63% of girls and 50% of boys have received at least one dose of HPV vaccine and ∼33% completed the series.^20^

The HPV vaccination rate for our sampled population was 7.3% (*n* = 14) for starting the series, 62.3% (*n* = 119) for completing the vaccine series, and 3.1% (*n* = 6) for not intending to complete the vaccine. Another small percent/population, 4.2% (*n* = 8), reported not knowing if they were vaccinated against HPV. Adolescent are not always aware of which vaccine was received since this is under the control of their parents' consent, may have been bundled with other required school-age vaccinations. Our completion rate is higher than the national average. The vaccine is currently a two-dose regimen, a shift from a three-dose regimen in 2016 offering protection to those not completing the HPV vaccine series. Specifically, a two-dose regimen is recommended for girls and boys starting the HPV series at ages 9 through 14 years, and three doses remain for boys and girls starting the HPV series at ages 15 through 26 years. Among our study participants, vaccination rates were not separated by gender; the study population was largely (85.6%) female. Knowledge with regard to required immunization may be higher for college students due to vaccination college entrance requirements.^30^ The American College Health Association guidelines requirements for vaccine-preventable diseases policy implementations found that the immunization compliance rate among colleges were about 93% among sampled colleges.^30^

This study occurred in a state with requirements for some vaccination; Florida does no mandate HPV vaccination. A large majority of our sample (74%) of the younger age group held the belief of HPV vaccine being mandatory for children ages 9–12 years. This majority also strongly disagreed with statements that went against the importance of vaccinations. This could be a positive predictor of support of childhood vaccination for our future generation of parents.

Due to the immune system, HPV has a higher clearance rate among younger age. But a greater propensity for disease due to lower immune system has been reported with increasing age. The small percentage of 7.1% reported ever having a positive HPV diagnosis supports national guidelines indicating that HPV screening should not occur until age 30 years and above. The 26 years and older age group was more likely to agree with the statement that HPV was not harmful. This finding may indicate a need for greater health education regarding HPV and HPV vaccination. As reported in two studies, there was a significant relationship between receiving health information through technology and age.

Younger study participants were more likely to want HPV information through technology when compared with older study participants.^31,32^ Digital health technology can be cost effective and easily evaluated for effectiveness in facilitating HPV knowledge and immunization uptake. Opportunities to design age- and population-specific education interventions has a role in digital technology health information dissemination.

## Conclusion and Future Implications

Research findings supporting digital technology platforms to disseminate evidence-based HPV data and HPV vaccination educational information targeting adolescents and young adults in academic and other setting can help health care providers make informed decisions about age-appropriate, topic, and content-relevant information specifically targeting this population. The findings of this study indicated that work is needed in the acceptance of social media, SMS text messaging, and mobile Apps as preferred digital health technologies beyond the internet. Health care providers can educate parents and students with regard to navigating the health system to obtain health information by using text messages and mobile apps, easily and effectively. Electronic health records, patient portals, and local health departments are all possible repositories of vaccine information/status. Taking advantage of patient encounters, such as annual Well-Women Visits, provide opportune time to screen for STDs, update vaccinations, and provide sexual and reproductive health education. For example, Chlamydia is the most common reportable STD in the United States. HPV is the most common STD in women. Populations most burdened by STDs are adolescents and young adults ages 15–24 years. As per the CDC national guidelines, sexually active women under the age of 25 years should be screened for gonorrhea, an opportune time to discuss HPV and HPV vaccination.^1^ HPV testing is not recommended in this age group, not until 30 years of age.^33^ However, CDC, Association of American Cancer Institutes, and American Cancer Society—leaders in HPV cancer prevention—have partnered to establish an HPV Vaccination Champion to reinforce that HPV Vaccine is Cancer Prevention.^34^

The HPV Vaccination Roundtable is one such Vaccine Champion developing the campaign “We're In!” a Facebook profile picture frame.^34^ Facebook picture frames are a way individuals can demonstrate support for HPV vaccination as cancer prevention.^34^ Facebook is one of many digital health platforms providing evidence-based health information similar to Twitter, Instagram, Google+, Vimeo, YouTube, Digg, Flikr, and Pinterest. Internet or online websites are the preferred digital health technologies for these study populations. Growing evidence support using digital health technologies to inform decision making to increase HPV risk perception and promote HPV vaccine uptake among adolescents and young adults. Digital health technology beyond the internet and online websites is warranted and when used appropriately can be a strategic and successful tool to inform decision making, increase perception, and promote HPV vaccine uptake. Text messaging can be used to receive updates from healthcare providers.

## Abbreviations Used

CDC: Centers for Disease Control and Prevention
FDA: Food and Drug Administration
HBM: health belief model
HPV: human papillomavirus
SMS: short message service
STD: sexually transmitted disease
STI: sexually transmitted infection
